# Hedonic sensitivity to low-dose ketamine is modulated by gonadal hormones in a sex-dependent manner

**DOI:** 10.1038/srep21322

**Published:** 2016-02-18

**Authors:** Samantha K. Saland, Kristofer J. Schoepfer, Mohamed Kabbaj

**Affiliations:** 1 Department of Biomedical Sciences, Program in Neurosciences, College of Medicine, Florida State University, USA.

## Abstract

We recently reported a greater sensitivity of female rats to rapid antidepressant-like effects of ketamine compared to male rats, and that ovarian-derived estradiol (E2) and progesterone (P4) are essential for this response. However, to what extent testosterone may also contribute, and whether duration of response to ketamine is modulated in a sex- and hormone-dependent manner remains unclear. To explore this, we systematically investigated the influence of testosterone, estradiol and progesterone on initiation and maintenance of hedonic response to low-dose ketamine (2.5 mg/kg) in intact and gonadectomized male and female rats. Ketamine induced a sustained increase in sucrose preference of female, but not male, rats in an E2P4-dependent manner. Whereas testosterone failed to alter male treatment response, concurrent administration of P4 alone in intact males enhanced hedonic response low-dose ketamine. Treatment responsiveness in female rats only was associated with greater hippocampal BDNF levels, but not activation of key downstream signaling effectors. We provide novel evidence supporting activational roles for ovarian-, but not testicular-, derived hormones in mediating hedonic sensitivity to low-dose ketamine in female and male rats, respectively. Organizational differences may, in part, account for the persistence of sex differences following gonadectomy and selective involvement of BDNF in treatment response.

Beyond the well-established female preponderance in depressive disorders^1^,^2^, sex differences have also been identified in antidepressant efficacy that suggest a role for gonadal hormones in moderating treatment response^3^,^4^. Despite its clear importance, the precise nature of hormonal influence on antidepressant efficacy is equivocal and understudied. This issue is secondary to a more fundamental problem concerning the stagnant progress in development of more efficacious medications for the treatment of depression and other mental health disorders^5^,^6^. It is therefore understandable why significant excitement has been generated by the recent discovery that the N-methyl d-aspartate receptor (NMDAR) antagonist, ketamine, produces rapid relief of depressive symptoms in patients with treatment-resistant depression^7^,^8^.

We recently reported new evidence of a greater sensitivity of female rats to the rapid antidepressant-like effects of low-dose ketamine (2.5 mg/kg) in the forced swim and novelty suppressed feeding tests when compared to male rats, and that ovarian-derived estrogen (E2) and progesterone (P4) are both required for this heightened response^9^. This work has since been validated in mice^10^. However, sex differences in baseline responding in these acutely-stressful behavioral paradigms can interfere with the ability to tease apart hormone-dependent contributions to ketamine’s response profile from those consequent to sex differences in response to stress, environmental variables and other factors. In addition, these behavioral assays were developed to detect the efficacy of drugs with similar mechanisms of action to first-generation prototype drugs^6^, and therefore yield little benefit for identification of novel therapeutic targets for newer drugs with distinct mechanisms of action. Because we still know so little about the proximal effects mediating ketamine’s rapid and sustained therapeutic efficacy, combined with the already complex actions of gonadal- and brain-derived hormones, a more fruitful approach may be achieved through use of more translationally-relevant behavioral components with well-known circuitry, where subjects can be repeatedly measured over time.

Anhedonia, the decreased ability to experience pleasure from or desire for normally pleasurable activities, is one symptom common to several mental health illnesses that may serve as a tractable intermediate phenotype for drug discovery^11^. There is a substantial knowledge of reward circuitry, and these circuits appear to be reasonably well-conserved between rodents and humans^6^,^12^,^13^. Clinically significant anhedonia is experienced by significant proportion of patients with major depressive disorder^14^, and is a predictor of poorer treatment prognosis relative to their non-anhedonic counterparts^15^,^16^,^17^. Excitingly, while currently available antidepressants are generally ineffective in alleviating anhedonia, ketamine has recently been shown to relieve anhedonic symptoms in both rodents^9^,^18^,^19^ and human patients with depression^20^,^21^. As such, preclinical tests assessing hedonic behavior are an ideal starting point for teasing apart the influence of hormonal status on the differential sensitivity of male and female rodents to the antidepressant-like actions of low-dose ketamine. This strategy falls in line with the recent National Institute of Mental Health Research Domain Criteria (RDoC) initiative, which proposes a dimensional approach to diagnostic criteria for mental health disorders that implicate specific emotional, behavioral and cognitive domains as the basis for understanding of such illnesses^22^.

Therefore, in this work we used a continuous-access sucrose preference test to evaluate whether activational and/or organizational effects of gonadal hormones underlie their contribution to the differential sensitivity of male and female rats to low-dose ketamine. Here, the effect of ketamine on hedonic behavior was systematically investigated in intact and gonadectomized adult male and female rats treated identically with physiologically-relevant doses of estradiol (E2), progesterone (P4), E2 + P4 or testosterone (T) (see Fig. 1 for experimental design). By collecting repeated measurements for each individual throughout the experiment, this mixed between-/within-subjects approach provides a highly sensitive measure of hedonic behavior on an individual basis, yielding high statistical power and simultaneous evaluation of hormone- and subject-specific predictors of treatment response. In addition, because the neurotrophic factor BDNF represents a major point of convergence in the hippocampus between known mechanisms of action of ketamine on antidepressant-like response^23^,^24^ and gonadal hormones in affective behavior^25^, we investigated whether alterations of this protein and its downstream signaling effectors were associated with hedonic-like response to low-dose ketamine in male and female rats with various hormonal profiles.

## Results

### Influence of cyclic E2 and P4 treatment on hedonic response to low-dose ketamine in ovariectomized female rats

Following a single injection of low-dose of KET (2.5 mg/kg), OVX female rats treated with both E2 and P4 showed a robust increase in sucrose preference relative to their SAL-treated levels, persisting for 7 days post-treatment (Fig. 2a; Treatment/Day: F_(8,352)_ = 5.134, p < 0.0001; Hormone: F_(3,44)_ = 2.531, p = 0.0693; Interaction: F_(24,352)_ = 1.839, p = 0.0103; p < 0.01; Supplementary Table S1 online). Conversely, sucrose preference was unaltered in OVX females treated with OIL, E2 or P4 alone (all p > 0.05), suggesting that both hormones are required to increase hedonic response to low-dose KET in this paradigm. Separate analyses of fluid and caloric intake confirmed that increased sucrose preference levels in E2P4-treated rats following ketamine were not secondary to changes in general consummatory behavior or metabolic needs (Supplementary Figure S1 online). No significant differences in baseline sucrose preference between groups were apparent (p > 0.05).

Simple linear regression analyses revealed that SAL baseline sucrose preference scores only predicted response to ketamine in E2P4-treated rats (Fig. 2e; F_(1,10)_ = 23.73, p = 0.0007, R^2^ = 0.7035), suggesting that the predictive ability of basal hedonic preference levels on treatment response in OVX females depend on hormonal status (Fig. 2b–d; OIL: F_(1,8)_ = 0.7167, R^2^ = 0.08222; E2: F_(1,12)_ = 0.3812, R^2^ = 0.03079; P4: F_(1,10)_ = 0.2293, R^2^ = 0.02242; all p’s >0.05). Results from all statistical analyses are reported in Supplementary Table S1 online.

### Effect of low-dose ketamine on hedonic behavior following cyclic E2 and P4 treatment in intact male rats

When intact male rats were administered cyclic hormone treatment identical to that used in OVX females, KET induced a long-lasting increase in sucrose preference of P4-treated rats (all p < 0.05; Supplementary Table S2 online), but was without effect (p > 0.05) in any other treatment group (Fig. 3a; Treatment/Day: F_(8,272)_ = 3.939, p = 0.0002; Hormone: F_(3,34)_ = 8.386, p = 0.0003; Interaction: F_(24,272)_ = 2.195, p = 0.0014). Further analysis of general consummatory behavior (Supplementary Figure S2 online) revealed that the pro-hedonic response to ketamine observed in P4-treated males was not simply due to changes in fluid or caloric intake. Overall weight gained throughout the study by E2- and E2P4-administered intact male rats was substantially lower than that of their OIL-treated counterparts (Fig. 3f; E2, E2P4: p < 0.0001; F_(3,34)_ = 28.86, p < 0.0001), which was associated with comparatively lower sucrose preference (E2: q_(34)_ = 2.591, p = 0.0363 vs. OIL; E2P4: q_(34)_ = 3.172, p = 0.0086 vs. OIL) and caloric intake, and higher levels of water consumed (Supplementary Figure S2 online), before and after KET treatment. However, lower baseline preference levels and calories consumed do not likely account for the lack of hedonic response to KET in these animals, supported by the lack of positive correlation between sucrose preference and consummatory fluctuations for all treatment groups across the post-treatment period (see Supplementary Figure S2 online).

Interestingly, SAL baseline preference levels were highly predictive of magnitude of response to KET in OIL-, E2- and P4-treated, but not E2P4-treated, intact male rats (Fig. 3b–e; OIL: F_(1,6)_ = 60.51, p = 0.0002 R^2^ = 0.9098; E2: F_(1,8)_ = 15.13, p = 0.0046, R^2^ = 0.6541; P4: F_(1,8)_ = 251.7, p < 0.0001, R^2^ = 0.9692; E2P4: F_(1,8)_ = 1.272, p = 0.2921, R^2^ = 0.1372). However, regression scatter plots show roughly equivalent numbers of data points falling above and below the 100% baseline indicator in OIL- (Fig. 3b) and E2-treatment (Fig. 3c) groups. Here, rats with higher baseline sucrose preferences (>70%) showed reduced hedonic response to KET, whereas increased responses were observed in those with lower (<70%) preferences. Therefore, baseline sucrose preference predicts both positive and negative treatment response in OIL- and E2-treated male rats, whereas the magnitude of positive response is predicted in P4-treated rats. Results from all statistical analyses are presented in Supplementary Table S2.

### Effect of chronic testosterone treatment on hedonic response to low-dose ketamine in intact female rats

To determine whether activational effects of testosterone might reduce sensitivity to low-dose KET, we first administered testosterone or cholesterol placebo chronically to intact adult female rats. After receiving a low-dose injection of KET, placebo-treated female rats displayed heightened sucrose preference levels that fluctuated throughout the 7 days following treatment (Fig. 4a; p < 0.05, Supplementary Table S3 online). In stark contrast, KET had no effect in female rats with testosterone pellet implants (Fig. 4a; Treatment/Day: F_(8,144)_ = 3.390, p = 0.0014; Hormone: F_(1,18)_ = 6.689, p = 0.0172; Interaction: F_(8,144)_ = 1.289, p = 0.2539). As sucrose intake was similar between groups, this discrepancy was largely attributed to the greater volumes of water consumed by testosterone-administered female rats, both prior to and following KET treatment, relative to those receiving placebo (Supplementary Figure S3 online). Unlike findings reported above, KET-induced reductions in water and caloric intake were either absent (Intact + P) or mild (Intact + T) in both groups, and neither fluid nor caloric intake positively correlated with preference scores across the post-treatment period, discounting the influence of altered consummatory behavior or energy requirements in determining hedonic response to KET (see Supplementary Figure S3 online).

Both baseline preference (F_(1,8)_ = 44.05, p = 0.0002, R^2^ = 0.8463) and sucrose intake levels (Supplementary Figure S3 online; F_(1,8)_ = 14.74, p = 0.0050, R^2^ = 0.6482) were strongly associated with the magnitude of hedonic response to KET in placebo-treated females, but not those receiving testosterone pellets (Fig. 4b,c; p > 0.05). Here, a greater positive response to KET was observed in animals consuming lower quantities of sucrose solution prior to treatment. Neither water consumption, caloric intake nor body weight correlated with treatment response in either group (p > 0.05).

Given the co-requirement of E2 and P4 in female rats for pro-hedonic effects of low-dose KET in this behavioral paradigm (Experiment 1), estrous cycles were continuously monitored by vaginal lavage to account for potential hormone- or injection-stress induced disruptions. As portrayed in Fig. 4e, chronic testosterone treatment led to sustained disruption of estrous cyclicity in intact female rats. Persistent diestrus smears were evident from 10–14 d post-surgery throughout experiment’s entirety, shown by the continuous presence of sparsely-packed leukocytes and, in some females, cornified epithelial cells. Disruption by testosterone, rather than injection stress, is supported by the maintenance of normal 4–5 d cycles in placebo-treated rats. Notable physiological alterations observed in Intact +T females, including greater caloric intake and body weight gain (Fig. 4d; Welch’s t-test: t_(17.08)_ = 3.869, p = 0.0012 vs. Intact + P), are consistent with absent or abnormal E2/P4 fluctuations. Results from all statistical analyses are presented in Supplementary Table S3 online.

### Effect of low-dose ketamine on hedonic behavior following gonadectomy and testosterone supplementation in male rats

Despite having enduring effects on general consummatory behavior (Supplementary Figure S4 online), depletion of gonadal testosterone in adult male rats had no effect on sensitivity to hedonic actions of KET. As expected, low-dose KET failed to elicit any effect on sucrose preference in males above and beyond normal daily fluctuations, regardless of hormonal status (Fig. 5a; Treatment/Day: F_(8,216)_ = 3.577, p = 0.0006; Hormone: F_(2,27)_ = 6.995, p = 0.0036; Interaction: F_(16,216)_ = 1.093, p = 0.3627). A gradual reduction in water intake in placebo-treated GDX rats reached significance + 6d post-treatment, and modest increases in sucrose consumed on the day of KET treatment were exhibited by both SHAM and GDX + T male rats (Supplementary Figure S4 online); however, the brevity of this effect along with concurrent caloric intake elevations suggest a general rise in consummatory behavior, rather than pro-hedonic actions of KET, *per se* (Supplementary Figure S4 online).

Independent of treatment with KET, GDX and testosterone replacement induced robust effects on baseline measures for all parameters considered. Gonadal hormone depletion significantly reduced sucrose preference (Fig. 5a; p < 0.05 vs. SHAM, GDX + T; see Supplementary Table S4 online), consumption (p < 0.01 vs. SHAM, GDX + T) and caloric intake (p < 0.0005 vs. SHAM, GDX + T) at baseline and throughout the post-treatment period (Supplementary Figure S4 online). Testosterone supplementation at the time of castration protected against development of these decrements, confirming robust efficacy of the chronic treatment regimen used herein. Despite the large behavioral distinctions between male rats deprived or not of peripheral testosterone supplies, levels of hedonic responding following KET administration were unrelated to baseline preference levels (Fig. 5b–d). A comprehensive list of results from all statistical analyses can be found in Supplementary Table S4 online.

### Integrated analysis of ketamine’s effects across sex and hormonal status: Z-score normalization of sucrose preference

Post-treatment sucrose preference measures were summarized across experiments to compare the hedonic response to low-dose KET under different hormonal conditions within each sex (Fig. 6). Individual sucrose preference scores were rescaled by expressing values as percent change from baseline, allowing within-sex comparison of treatment response in groups from independent experiments along the same scale. Data depicted in Fig. 6a,b represent group means collapsed across days of the post-treatment period. When compared in this manner, the same effects of KET are observed for both sexes. When compared to OVX + OIL controls, E2P4-treated OVX females (p = 0.0103) and intact females receiving placebo (p = 0.0222) display significant increases in sucrose preference relative to their SAL baseline levels (Fig. 6a; F_(5,60)_ = 4.958, p = 0.0007). Conversely, only P4-treated intact males (p = 0.0143) show a positive hedonic response relative to their OIL-treated counterparts (Fig. 6b; F_(6,61)_ = 5.256, p = 0.0002).

In order to identify the relative magnitude of response to KET between same sex-groups, data in Fig. 6a,b were standardized via z-score normalization within each sex, eliminating behavioral “noise” from repeated measures data by accounting for non-uniformity of variances between experimental cohorts. Z-score values are presented in Fig. 6c,d as the number of standard deviations of each group from their respective OIL-treated control means. In females, only cyclic E2P4 treatment in OVX rats (p = 0.01) restored the behavioral response to KET to levels similar to those observed for normally cycling females (Fig. 6c, Intact + P: p = 0.0222; F_(5,60)_ = 4.958, p = 0.0007), validating the treatment regimen used and emphasizing the requirement of both hormones in female rats for a pro-hedonic to low-dose KET. Interestingly, this regimen is ineffective in intact males; whereas treatment with only P4 (p = 0.0143) enhanced the sensitivity of males to this dose of KET, significantly increasing sucrose preference relative to OIL-treated males (Fig. 6d; F_(6,61)_ = 5.256, p = 0.0002). By correcting for sex differences in basal preference levels (Fig. 6e), we found that the magnitude of the P4-mediated response to KET in males (p = 0.0301) was similar to that of intact (p = 0.0057) and E2P4-treated OVX (p = 0.0018) females F_(12,121)_ = 4.551, p < 0.0001).

### Effect of cyclic E2 and P4 treatment on hippocampal BDNF protein levels and downstream signaling effectors in female and male rats

Protein levels of BDNF were substantially increased in the dorsal hippocampus of E2P4-treated OVX female rats 24 h following an acute low dose of KET relative to OIL-treated controls (*p* = 0.0345), but were unaltered (*p* > 0.05) in those receiving cyclic E2 or P4 alone (Fig. 7a; F_(3,20)_ = 1.574, p = 0.0004). Conversely, BDNF levels were unaffected by ketamine in treatment-responsive intact male rats treated with cyclic P4 (p > 0.05), and showed decreases in E2- (*p* = 0.0006) and E2P4-treated (*p* = 0.0034) male rats when compared to OIL-treated controls (Fig. 7b; F_(3,20)_ = 1.385, p < 0.0001). A significant positive correlation was observed between BDNF levels and sucrose preference (average percent change from baseline across the post-treatment period) for E2P4-treated OVX females (data not shown; *p* = 0.0036, R^2^ = 0.9034), but not for any other group of OVX females or intact males (*p* > 0.05).

Phosphorylation of key proteins within three primary downstream BDNF-TrkB signaling pathways was next evaluated to identify potential mechanisms contributing to, or resulting from, sex-dependent involvement of hippocampal BDNF protein in treatment response. Regardless of hormone regimen, levels of total (Females: F_(3,20)_ = 1.154, p = 0.3517; Males: F_(3,20)_ = 0.2424, p = 0.8657) and phosphorylated (Females: F_(3,20)_ = 1.122, p = 0.3640; Males: F_(3,20)_ = 2.440, p = 0.0943) hippocampal AKT protein were similar (*p* > 0.05) between ketamine-responsive and non-responsive groups of male and female rats 24 h following treatment (Fig. 7c,d).

An effect of hormone treatment on ERK phosphorylation was observed in females (Fig. 7e), with greater levels of p-ERK detected in OVX rats receiving E2 alone (p = 0.0047), but not those receiving P4 or E2P4 (p > 0.05), when compared to OIL-treated controls (F_(3,20)_ = 4.503, p = 0.0143). No differences in total ERK abundance were found between treatment groups in female rats (F_(3,20)_ = 1.516, p = 0.2409). Conversely, neither total levels of ERK1/2 (F_(3,20)_ = 2.571, p = 0.0829) nor its phosphorylation status (Fig. 7f; F_(3,20)_ = 0.7044, p = 0.5605) were affected by hormone treatment in male rats (Fig. 7f).

Examination of hippocampal CaMKIIα protein expression by western blot revealed distinct sex-dependent patterns of regulation by hormone treatment in female and male rats (Fig. 7g,h). While similar levels of both phosphorylated and total CaMKIIα protein were observed between E2P4- and OIL-treated female rats (p > 0.05), non-treatment responsive OVX females receiving either E2 (p = 0.0033) or P4 (p < 0.0001) alone exhibited reduced levels of total, but not phosphorylated, CaMKIIα relative to their OIL-treated counterparts (Fig. 7g; total: F_(3,20)_ = 16.45, p < 0.0001, phospho: F_(3,20)_ = 1.936, p = 0.1563). Interestingly, levels of both phosphorylated and total CaMKIIα were detected in E2- (total: p = 0.0196; phospho: p < 0.0001) and E2P4- (total: p = 0.0017; phospho: p < 0.0001) treated, but not P4-treated, male rats compared to OIL-treated controls (Fig. 7h; total: F_(3,20)_ = 7.282, p = 0.0017, phospho: F_(3,20)_ = 18.51, p < 0.0001).

## Discussion

The present study is the first of its kind to systematically investigate the nature of gonadal hormone influence on the differential sensitivity of male and female rats to low-dose ketamine in the context of hedonic behavior, as well as the therapeutic potential of these hormones as adjuncts to enhance the effectiveness of ketamine at suboptimal doses. As expected, a single low dose of ketamine (2.5 mg/kg) selectively enhanced sucrose preference of female rats in an E2P4-dependent manner, with no effect in males, confirming selective enhancement of female responsivity to this dose reported in our previous work^9^. In extension, this hormone-mediated effect was protracted, lasting up to 7 days. Of note was the finding that cyclic treatment with P4 alone, but not E2P4, significantly enhanced hedonic response of intact male rats to the same low dose of ketamine. Furthermore, positive treatment response was associated with increased BDNF protein levels in the dorsal hippocampus of female rats only, suggesting that hedonic response to ketamine and its modulation by sex steroids in male and female rats are mediated via distinct mechanisms. Collectively, these findings provide novel evidence supporting both activational and therapeutic roles for ovarian-, but not testicular-, derived hormones in mediating hedonic sensitivity to ketamine in both sexes, and suggest potential therapeutic implications for progesterone or progesterone-like compounds as adjunctive treatments in males.

In extension of our previous work^9^ and supporting evidence recently demonstrated in mice^10^, we first sought to confirm the E2P4-dependent enhancement of female rats to low-dose ketamine, using hedonic behavior as the dependent variable of interest. A continuous-access sucrose preference paradigm was used in order to investigate both the magnitude and duration of response induced by ketamine. In agreement with our original report^9^, a single low dose of ketamine significantly enhanced sucrose preference above saline-treated levels in E2P4-treated OVX female rats, without effect in OIL-, E2- or P4-treated OVX rats. These effects were not secondary to changes in fluid or caloric intake, confirming that low-dose ketamine selectively enhanced hedonic valence in female rats in the presence of both E2 and P4. Interestingly, baseline sucrose preference levels only predicted the magnitude of response to ketamine in E2P4-treated OVX female rats, suggesting that the predictive ability of baseline hedonic valence on the magnitude of response to ketamine in OVX females is dependent on hormonal status. Here, a greater enhancement of hedonic response was observed in animals with lower baseline preference levels.

These findings suggest that the influence of E2 and P4 on enhanced sensitivity to ketamine in females are, at least in part, activational in nature. Therefore, we administered identical cyclic hormone treatment regimens to intact male rats in order to determine whether these hormones might increase their sensitivity to low-dose ketamine. Interestingly, cyclic treatment with P4 alone was sufficient to enhance ketamine’s efficacy in intact males, significantly increasing sucrose preference levels for up to one week. Neither fluid nor caloric intake could explain the increased preference levels across the post-treatment period. Conversely, ketamine was without effect in OIL-, E2-, and E2P4-treated male rats. While both E2- and E2P4-treated male rats both gained significantly less weight throughout the course of the experiment, their sucrose preference levels following ketamine treatment were unaffected by overall fluid or caloric intake. In contrast to observations in OVX female rats, saline baseline preference levels were highly predictive of magnitude of response to ketamine in OIL-, E2- and P4-treated, but not E2P4-treated, intact male rats. However, the magnitude of *positive* response was predicted by baseline sucrose preference only in treatment-responsive groups of female (OVX + E2P4) and male (Intact + P4) rats, whereas baselines of non-responsive OIL-, E2- and E2P4-treated intact males were predictive of response in both directions, as reflected by the similar number of points falling above and below baseline in the regression scatterplots.

An alternative hypothesis for the sex-dependent sensitivity to ketamine is that testosterone may reduce responsivity in males. To address this possibility, hedonic effects of low-dose ketamine were investigated in sham-operated (SHAM) and gonadectomized (GDX) adult male rats receiving either placebo (GDX + P) or testosterone pellet (GDX + T) supplementation. While gonadectomy induced an anhedonic-like state in male rats, ketamine failed to alter sucrose preference levels in all males, regardless of baseline preference or circulating testosterone levels. As well, negligible effects of this drug were observed on fluid or caloric intake throughout the post-treatment period. The lack of effect of ketamine in these animals across all parameters measured strongly supports than an organizational sex difference is involved in the differential sensitivity of male and female rats to ketamine.

To confirm this hypothesis, we examined whether chronic supplementation of the same dose of testosterone altered ketamine’s efficacy in intact female rats. Confirming our earlier findings, intact female rats exhibited a protracted enhancement of hedonic behavior following a single injection of ketamine. It is worth noting that this effect was not as robust as those observed in E2P4-treated OVX female rats, likely due to differences in estrous cycle stage between subjects at the time ketamine was administered. As observed in OVX + E2P4 rats, lower baseline sucrose preference levels in cycling female rats predicted a greater increase in hedonic response to ketamine. Interestingly, chronic testosterone treatment significantly reduced sucrose preference levels in female rats prior to treatment, relative to their own baseline preference levels and to that of normally cycling female rats, and completely prevented the pro-hedonic actions of ketamine observed in female rats. Of note, estrous cycles were persistently disrupted in all testosterone-treated females prior to and throughout the testing period. It is therefore likely that abnormal or absent fluctuations in ovarian hormone levels prevented treatment response, rather than a direct consequence of testosterone it self.

When comparing the effective hormone treatments and/or treatment-responsive conditions in both intact and gonadectomized male and female rats, the present data support the original hypothesis that activational effects of ovarian, rather than testicular, hormones primarily mediate the enhanced sensitivity of female rats to low-dose ketamine; however, organizational differences may, in part, account for the persistence of sex differences following gonadectomy in male rats. Specifically, the requirement of P4 for pro-hedonic response to low-dose ketamine in both sexes suggests a primary activational effect of this hormone. It seems likely that the co-requirement of E2 in females reflects an organizational difference between male and female rats, where P4-mediated events act through substrates available only in the context of a preceding E2 surge that prime the physiological environment necessary for it to act. Together, these findings provide the first evidence of robust and protracted enhancement of hedonic responsivity to low-dose ketamine by adjunctive hormone treatment.

The neurotrophic factor BDNF represents a major point of convergence in the hippocampus between known mechanisms of action of ketamine on antidepressant-like response^23^,^24^ and gonadal hormones in affective behavior^25^. As such, we examined levels of BDNF as well as phosphorylation of key proteins within three primary downstream BDNF-TrkB signaling pathways to identify potential mechanisms mediating the enhancement of hedonic-like response to low-dose ketamine in E2P4-treated female and P4-treated male rats. Interestingly, results demonstrated a sex- and hormone-dependent effect of ketamine on BDNF protein levels in the dorsal hippocampus. Here, a significant increase in BDNF was observed in the hippocampus 24 h following ketamine treatment in E2P4-treated OVX female rats, but not in male rats receiving P4, relative to their OIL-treated counterparts. This selective increase of hippocampal BDNF in treatment-responsive female rats was not associated with alterations in phosphorylation status of either ERK1/2, AKT or CaMKIIα at this timepoint, suggesting that these signaling effectors downstream of BDNF-TrkB activation may have contributed to, rather than resulted from, increased BDNF secretion and/or translation. These findings are consistent with those of Duman and colleagues (2010), demonstrating a transient increase in ERK and AKT phosphorylation which returned to baseline within 2 hours of acute ketamine administration^19^.

It is interesting to note that hippocampal BDNF protein levels were reduced in E2- and E2P4-treated male rats when compared to OIL-treated males, accompanied by corresponding reductions in total CaMKII abundance. These results parallel the significantly lower sucrose preference scores observed in E2- and E2P4-treated male rats prior to and following ketamine treatment. Specifically, OIL- and P4-treated rats show similar raw sucrose preference scores and levels of BDNF and CaMKII both before and after ketamine treatment, compared with the substantially lower sucrose preference and protein levels displayed by E2- and E2P4-treated males at the same timepoints. Based on these observations, hippocampal BDNF and CaMKII levels in E2/P4-treated male rats may reflect changes associated with hormonal modulation of baseline hedonic behavior, independent of treatment response. Nonetheless, it appears that the role of BDNF translation and/or release in mediating the heightened sensitivity of female rats to the pro-hedonic effects of ketamine is sex-dependent and may reflect underlying organizational differences in both ketamine’s mechanisms of action, as well as in the activational effects of estradiol and progesterone within the hippocampus. This is supported by the significant correlation between hippocampal BDNF levels and change in sucrose preference following ketamine in treatment-responsive E2P4-treated OVX animals only—a relationship absent in males and non-responsive females. It should not be discounted, however, that ovarian hormone-treated females used in this study were in a gonadal hormone-deprived anhedonic state, whereas males receiving the same treatment were not—it is therefore possible that a ceiling effect may account, in part, for the lack of effect of ketamine on BDNF protein levels in treatment-responsive P4-treated male rats.

Interactions between sex, hormones and environment generate significant complexity that makes it difficult to isolate independent contributions of any of these factors to behavioral outcomes, either at baseline or in response to a drug—this is particularly true under conditions of stress^26^. Therefore, we sought to reduce as many sources of this complexity as possible in order to first understand how gonadal hormones influence response to ketamine under non-stressful conditions in a sex-specific manner. Our choice of sucrose preference as a behavioral readout was guided by the extensive knowledge of circuitries mediating reward-related behavior^6^—which is reasonably well-conserved across species^6^—and the ability of hedonic behavior to be easily modeled in rodents^27^. Reward can be further subdivided into several measurable components that include consummatory “liking” (hedonic impact), “wanting” (motivation/anticipation for reward), and “learning” (reward representation and prediction)^12^,^27^.

The continuous-access sucrose preference paradigm employed in the present experiments reflect hedonic “liking”—a fundamental experience of pleasure reflecting the hedonic valence of a stimulus^27^—and lends several advantages to the interpretation and impact of our findings. Most importantly, continuous measurement in the homecage environment permitted assessment of stable baseline, or trait, hedonic behavior for each animal over time in a non-stressful environment, devoid of confounds introduced by reactivity to novel testing environments. By collecting continuous measures for each individual, “state” changes in hedonic behavior following ketamine administration in the present results can be directly attributed to treatment effects (relative to vehicle), rather than artifacts of variability over time. The within-subjects component of this design yielded more sensitive outcome measures by allowing each animal to serve as their own control, and by the ability to account for normal intra-individual variability over time. Substantial statistical power is generated by this type of repeated measures analysis, generating more sensitive data with fewer animals needed per group. In addition, confounding influences on the interpretation of outcome measures was reduced by utilizing the within-subject comparison of treatment response and simultaneous measurements of fluid and food intake as predictors and covariates, respectively, rather than restricting them to independent analysis. Prediction of treatment response as a factor of baseline hedonic behavior and hormonal status has relevant translational implications.

Despite the aforementioned advantages, some limitations presented by this approach relevant to the interpretation and generalizability of the present findings should be acknowledged. As the present work was conducted entirely under non-stressful conditions, it is possible that potential behavioral and/or molecular responses to ketamine in non-treatment responsive animals may have been masked by ceiling effects in either domain. High baseline sucrose preference levels in intact rats, for example, could preclude any further increase in hedonic behavior following low-dose ketamine treatment. While this possibility cannot be excluded, that we still observed a pro-hedonic response to ketamine in intact female rats and P4-treated intact male rats not exhibiting anhedonic-like behavior supports a degree of sensitivity in our paradigm high enough to detect subtle treatment-induced changes regardless of stress exposure. Indeed, treatment-response occurred in both intact and gonadectomized animals, despite persistent reductions in sucrose preference—reflecting an anhedonic state—induced by gonadal hormone depletion in the latter.

It is also well-established that sex, gonadal hormones and environmental stress exert independent and interacting influences on depressive-like behaviors and antidepressant response. Sex differences in baseline FST behaviors, for example, have been consistently reported (albeit in conflicting directions)^28^,^29^. While estrous cycle effects in this behavior are generally small^30^,^31^,^32^, their impact on antidepressant response is considerably larger^32^,^33^. Additionally, work from our lab has demonstrated on numerous occasions the significant impact of gonadectomy and hormone replacement on depressive-like behavior and antidepressant response in the sucrose preference test, FST and NSFT^9^,^34^,^35^,^36^. Sex and hormone effects become further complicated when animals are first exposed to varying social and environmental stressors^10^,^35^, which may result in similar or distinct (even opposite) effects on behavior and within the brain^28^. With this in mind, it is unclear at this time whether the present findings may extend to other depression-relevant behaviors and brain regions in rodents under conditions of stress—particularly concerning the efficacy of P4 treatment on enhancement of response to low-dose ketamine. Given the complex roles within the nucleus accumbens (NAc) and ventral tegmental area (VTA) that BDNF plays in susceptibility and resiliency to stress-induced anhedonia^37^,^38^, as well as antidepressant response^37^, investigation of these brain regions would be a worthwhile avenue for further exploration of this work.

Collectively, the findings presented herein support a primary and essential role of E2 and P4 in mediating the enhanced sensitivity of female rats to ketamine. Among the most exciting of the present findings was the P4-mediated enhancement of ketamine’s actions in intact male rats, providing the first evidence of robust and protracted enhancement of hedonic response to low-dose ketamine by adjunctive hormone treatment. This sex-specific hormonal response profile and persistence of sex differences following gonadectomy in male rats also suggest a strong influence of organizational differences in the behavioral effects of ketamine, as well its underlying mechanisms—as supported by the selective increase in hippocampal BDNF in treatment-responsive female rats. With strong efforts currently dedicated to finding safer ways to maintain antidepressant response, this novel evidence has great implications for the use of ketamine as an antidepressant treatment in both men and women. In particular, these findings may be of high relevance for the development of effective antidepressants in women suffering from postmenopausal depression and other forms of hormone-related depressive states, in light of the critical roles ovarian-derived hormones serve in the enhanced sensitivity of female rats to low-dose ketamine. On a more fundamental level, the systematic identification of hormonal influence across sexes on “trait” hedonic behavior and “state” hedonic response to low-dose ketamine provide a good foundation for future antidepressant research development across a wide range of behavioral domains.

## Materials and Methods

### Animals

Adult male (250–270 g) and female (200–225 g) Sprague-Dawley rats (Charles River, Wilmington, MA) were pair-housed in 43 × 21.5 × 25.5 cm plastic cages and maintained on a 12 h:12 h light:dark cycle (lights on at 0700 h) in a temperature- and humidity-controlled room. Food and water were available *ad libitum* throughout the duration of the study, and all animal protocols were carried out in accordance with the NIH Guide for Care and Use of Laboratory Animals and approved by the Institutional Animal Care and Use Committee of Florida State University.

### Ovariectomy/gonadectomy

Ovariectomy, gonadectomy and sham surgeries were performed as previously described^9^,^34^,^35^ with the exception that isoflurane (4% for induction, 1–3% for maintenance) was used as an anesthetic (Butler Schein Animal Health, Dublin, OH). The non-steroidal anti-inflammatory drug meloxicam (1.0 mg/mL) was injected subcutaneously before and after surgery, and bupivacaine (0.25% solution; 0.4 mL/kg) was applied topically as an analgesic.

### Cyclic hormone treatment regimen and testosterone supplementation

Chronic testosterone (0 or 25 mg/pellet; Innovative Research of America, Sarasota FL) supplementation and cyclic administration of 17-β-estradiol benzoate (0 or 2 μg in 100 μL sesame oil, s.c.) and progesterone (0 or 500 μg in 100 μL sesame oil, s.c.) (Sigma, St. Louis, MO) were performed as previously described^9^,^34^,^35^. See Experimental Design for details.

### Estrous cycle monitoring

Estrous cycles were monitored continuously in intact female rats (Experiment 4) via daily vaginal lavage as previously described^39^. Cytological smears were used to assign stages as follows: diestrus 1 was characterized by the presence of leukocytes and clusters of cornified epithelial cells, diestrus 2 was characterized by a predominance of leukocytes in combination with larger rounded pavement cells, proestrus was characterized by moderate numbers nucleated epithelial cells, and estrus was defined by the predominance of cornified epithelial cells.

### Continuous-access sucrose preference test

The sucrose preference test consisted of a two-bottle choice paradigm^9^,^34^,^35^,^40^ administered continuously throughout the experiment, except during the one-week surgery recovery period. After a 5-day habituation to two bottles of water, rats were given access to two pre-weighed bottles, one containing water and the other 0.25% sucrose. The position of the sucrose solution was alternated with water every 24 hours to account for possible location preference. The bottles were weighed at 0900 h and 1800 h daily and the preference for sucrose over water was used as a measure of hedonic behavior. The concentration of sucrose was chosen based on the similar preference scores it produced between male and female rats prior to hormonal manipulation, thus minimizing potential sex bias introduced by unequal baseline measures. In addition, the almost negligible contribution of a 0.25% sucrose solution to daily caloric intake minimized the dependency of preference levels on general consummatory behavior. Importantly, providing continuous free access to both sucrose and water solutions permitted a more reliable assessment of treatment-induced changes in hedonic behavior by dissociating transient (or “state”) levels of sucrose preference from more stable underlying “trait” preference levels on an individual basis.

### Experimental design

#### Experiment 1a: Effect of cyclic E2 and P4 treatment on hedonic response to low-dose ketamine in ovariectomized female rats

**Figure 1:**
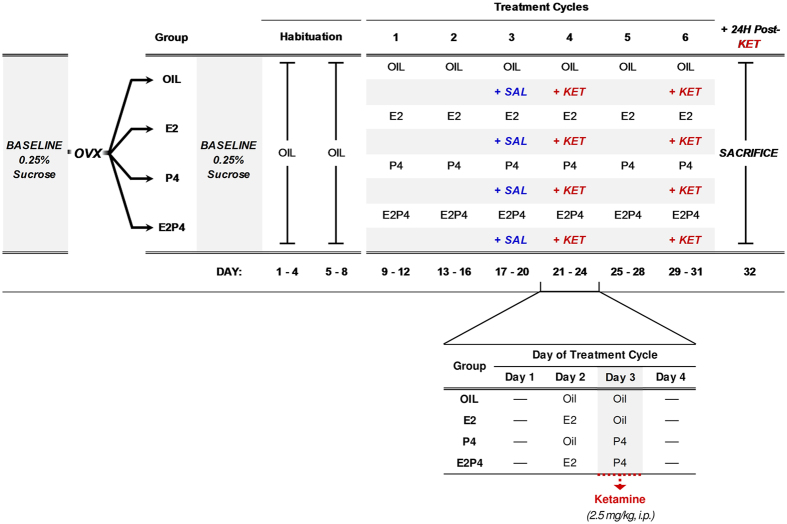
Timeline of procedures and cyclic hormone treatment regimen for Experiment 1. Abbreviations: E2, 17β-estradiol benzoate; KET, ketamine (2.5 mg/kg); OIL, sesame oil; OVX, ovariectomy; P4, progesterone; SAL, saline.

**Figure 2:**
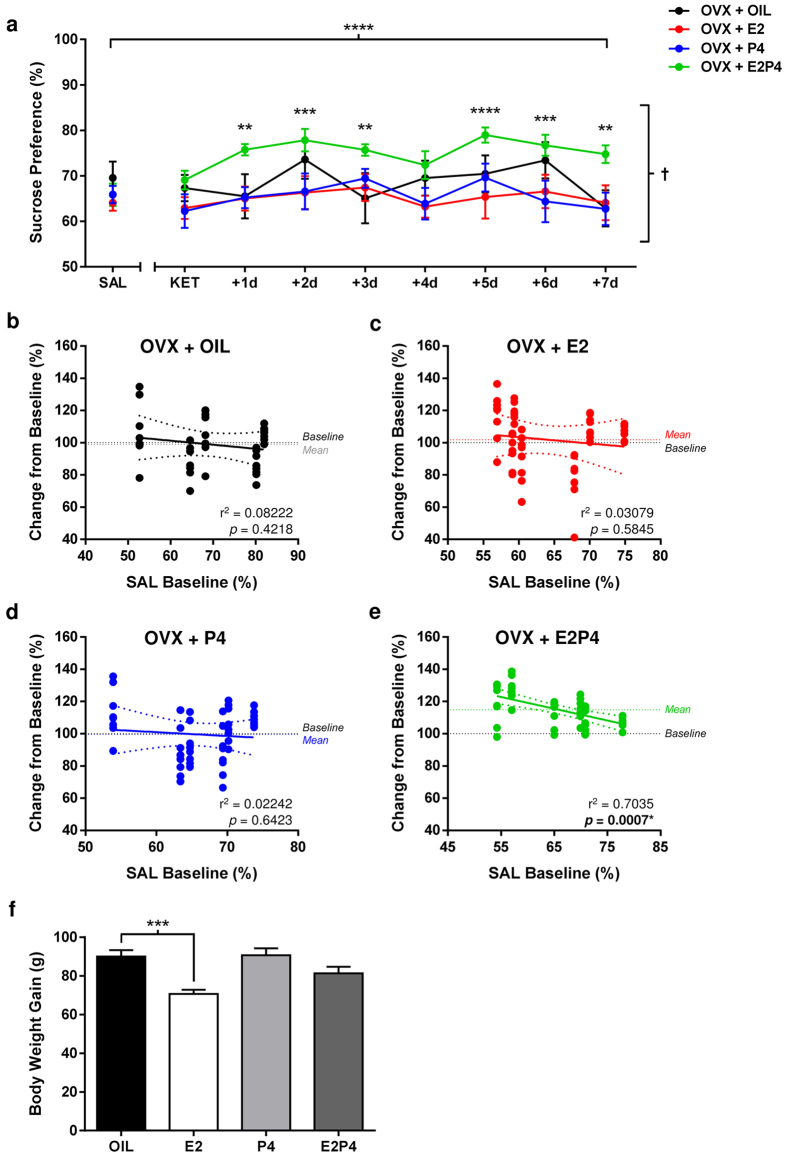
Estradiol and progesterone are required for rapid and sustained hedonic-like effects of low-dose ketamine in female rats. (**a**) Ketamine (KET, 2.5 mg/kg, i.p.) induced a significant and protracted increase in sucrose preference in ovariectomized (OVX) female rats treated with E2P4 (****p < 0.0001, ***p < 0.001, **p < 0.01 vs. SAL), but not OIL, E2 or P4 alone (Main Effects: Treatment/Day, ****p < 0.0001; Hormone: ^ߤ^*p* = 0.0693). Data are expressed as mean ± SEM (n = 48). (**b***–***e**) Saline (SAL) baseline sucrose preference levels predicted magnitude of positive response to KET in E2P4-treated OVX females only (r^2^ = 0.7035, *p* = 0.0007). (**f**) Significantly reduced overall body weight gain of E2-treated OVX female rats compared to OVX + OIL females (***P < 0.001) confirmed efficacy of hormone treatment. Data are expressed as mean ± SEM (n = 48).

**Figure 3:**
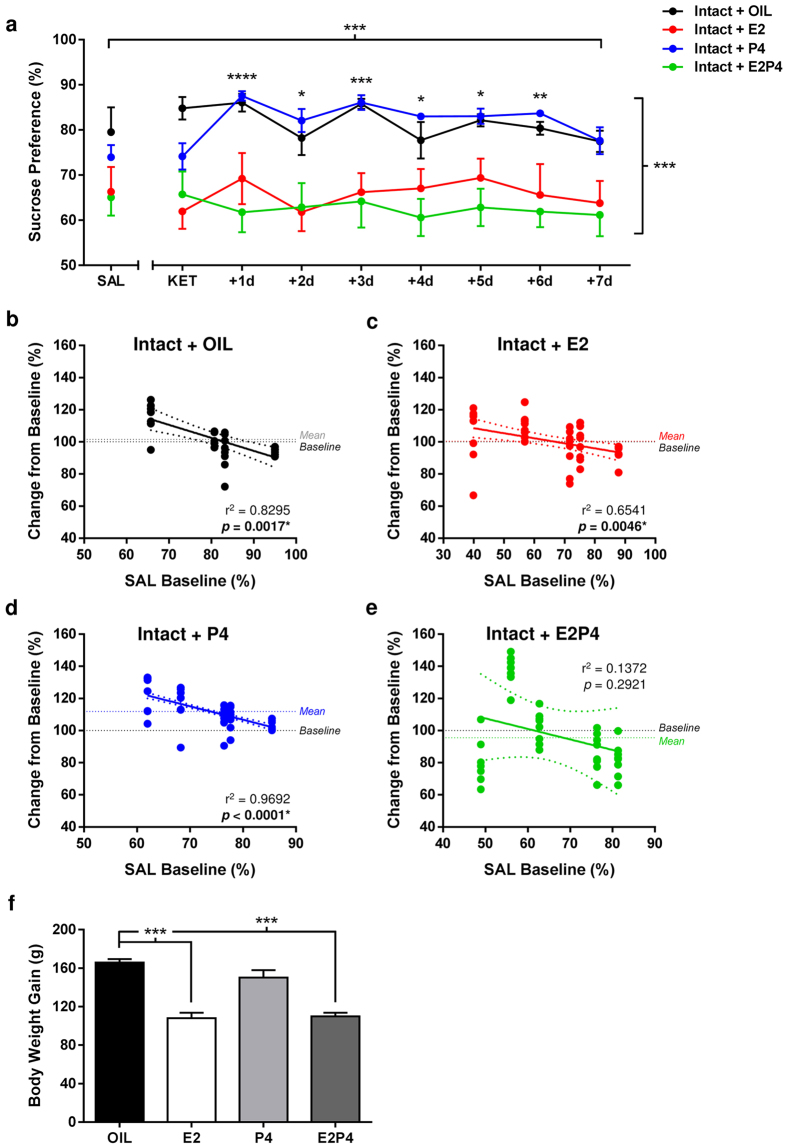
Cyclic P4 treatment enhances the hedonic sensitivity of intact male rats to low-dose ketamine. (**a**) Cyclic P4 treatment increased sensitivity of male rats to low-dose ketamine (KET) treatment (****p < 0.0001, ***p < 0.001, **p < 0.01, *p < 0.05 vs. SAL) for up to 6 days following drug administration (Main Effects: Treatment/Day, ***p < 0.001; Hormone: ***p < 0.001). Data are expressed as mean ± SEM (n = 40). (**b**–**e**) Lower saline (SAL) baseline sucrose preference levels predicted a higher magnitude of positive response to KET in treatment-responsive P4-treated intact males (r^2^ = 0.9692, *P* < 0.0001). (**f**) Cyclic treatment with E2 alone or in combination with P4 significantly reduced overall body weight gain of intact male rats compared to Intact + OIL males (***P < 0.001), confirming effective hormone treatment. Data are expressed as mean ± SEM (n = 38).

**Figure 4:**
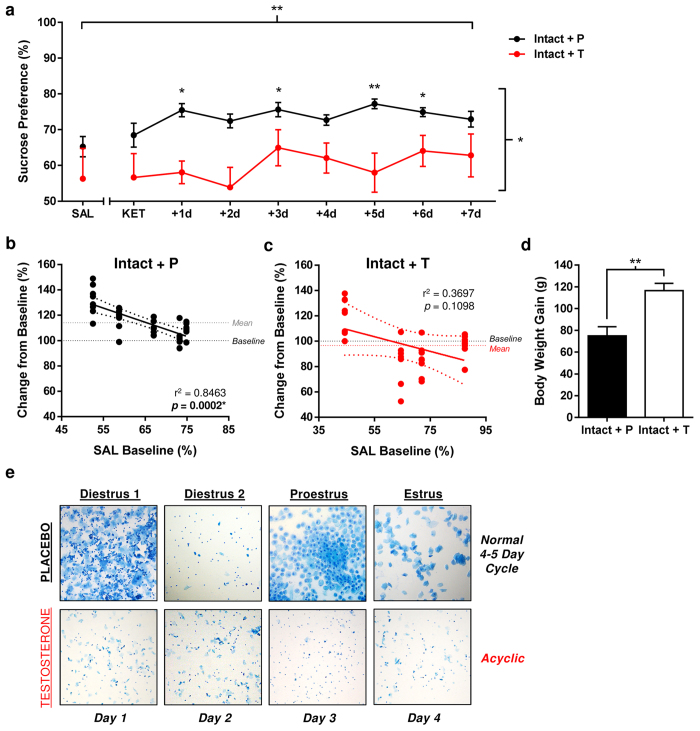
Chronic testosterone treatment blocks pro-hedonic like effects of ketamine in intact female rats via persistent disruption of estrous cyclicity. (**a**) Chronic testosterone treatment in intact female rats induced anhedonic behavior (**P < 0.005) and blocked response to ketamine (2.5 mg/kg, i.p.) (P > 0.05). Acute ketamine treatment led to a modest increase in sucrose preferences of placebo-treated intact female rats (**P < 0.01, *P < 0.05 vs. SAL) that persisted for 5 days (Main Effects: Treatment/Day, **P < 0.01; Hormone: *P < 0.05). Data are expressed as mean ± SEM (n = 18). (**b**,**c**) Lower SAL baseline sucrose preference levels predicted a higher magnitude of positive response to KET in treatment-responsive placebo-treated intact females (r^2^ = 0.8463, *P* = 0.0002), but not those receiving testosterone pellets (*P* > 0.05). (**d**) Chronic treatment with testosterone significantly increased overall body weight gain of intact female rats relative to intact females receiving placebo pellets (**P < 0.01). Data are expressed as mean ± SEM (n = 18). (**e**) Chronic testosterone treatment resulted in persistent disruption of estrous cyclicity in intact female rats.

**Figure 5:**
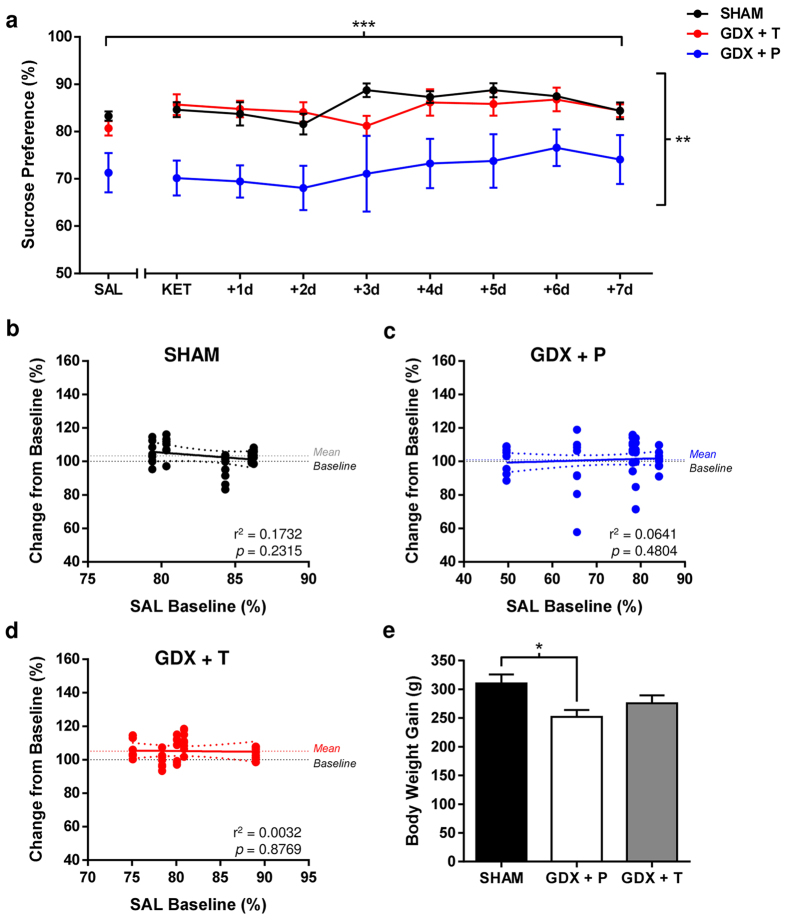
Gonadal testosterone does not influence sensitivity to low-dose ketamine in male rats. (**a**) Gonadectomized (GDX) male rats displayed a significantly lower sucrose preference when compared to SHAM & testosterone-supplemented male rats (**P < 0.01). Ketamine (KET; 2.5 mg/kg, i.p.) was without effect in male rats, regardless of hormonal status (Main Effects: Treatment/Day, ***P < 0.001; Hormone: **P < 0.01). Data are expressed as mean ± SEM (n = 30). (**b**–**d**) Saline (SAL) baseline preference levels of all males were not associated with magnitude of response to KET (*P* > 0.05). (**e**) Gonadal testosterone depletion resulted in significantly less body weight gain throughout the experiment relative to SHAM-operated males (*P < 0.05). Chronic testosterone supplementation at the time of gonadectomy was sufficient to block this effect (P > 0.05). Data are expressed as mean ± SEM (n = 30).

**Figure 6:**
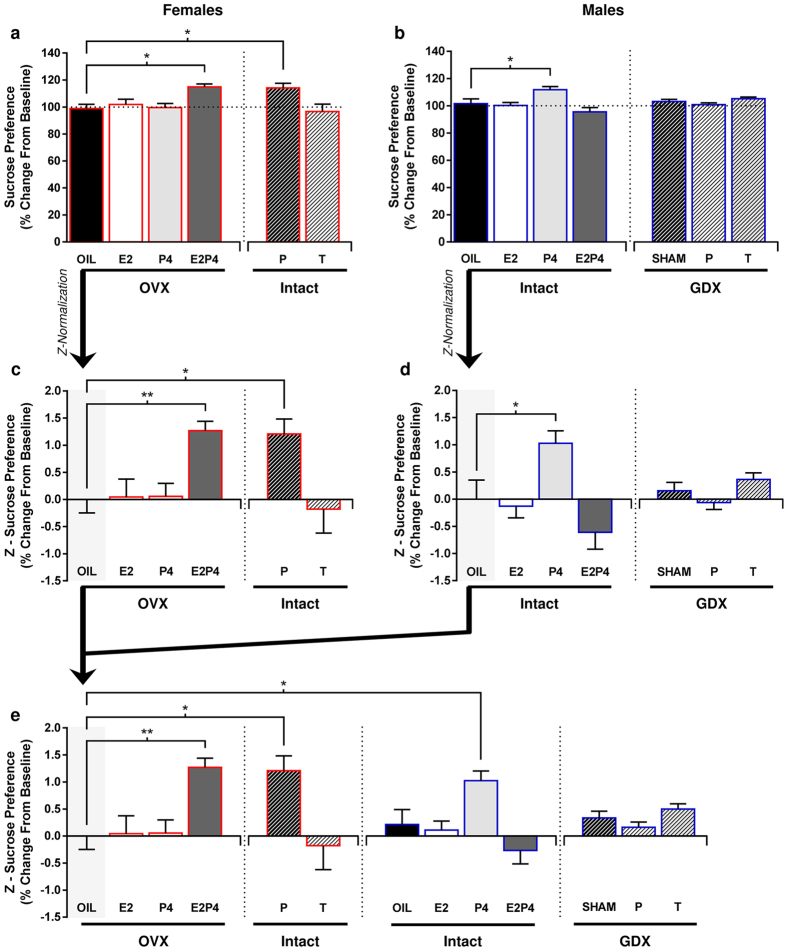
Integrated analysis of ketamine’s effects across sex and hormonal status. (**a**) Comparison of all groups of ovariectomized (OVX) and intact female rats demonstrating that normal cyclic fluctuation of both estradiol (E2) and progesterone (P4) levels are essential for pro-hedonic like response to low-dose ketamine (KET; 2.5 mg/kg) (*P < 0.05 vs. OVX + OIL). Data are expressed as mean ± SEM (n = 66). (**b**) Comparison of all groups of gonadectomized (GDX) and intact male rats reiterate the negligible effect of circulating testosterone (T) levels on male sensitivity to low-dose KET (P > 0.05), but identify effective enhancement of KET sensitivity in intact male rats by P4 treatment (*P < 0.05 vs. Intact + OIL). Data in (**a**,**b**) are presented as the percent change in sucrose preference following KET administration relative to saline (SAL) baseline levels, averaged across all days of the post-treatment period. Data are expressed as mean ± SEM (n = 68). (**c**,**d**) Standardization of female (n = 66) and male (n = 68) sucrose preference scores presented in (**a**,**b**), respectively, via Z-score transformation relative to OIL-treated groups of each sex (**P < 0.01, *P < 0.05). Data are expressed as mean ± SEM. (**e**) Percent change in sucrose preference levels from baseline following KET administration compared across sex and all hormone treatments via Z-score normalization of each group’s scores to OVX + OIL female rats. The magnitude of pro-hedonic like effects of KET was found to be similar in OVX + E2P4 (**P < 0.01) and Intact + P (*P < 0.05) female and P4-treated intact males (*P < 0.05) when controlling for unequal variances between all experimental cohorts. Data are expressed as mean ± SEM (n = 134).

**Figure 7:**
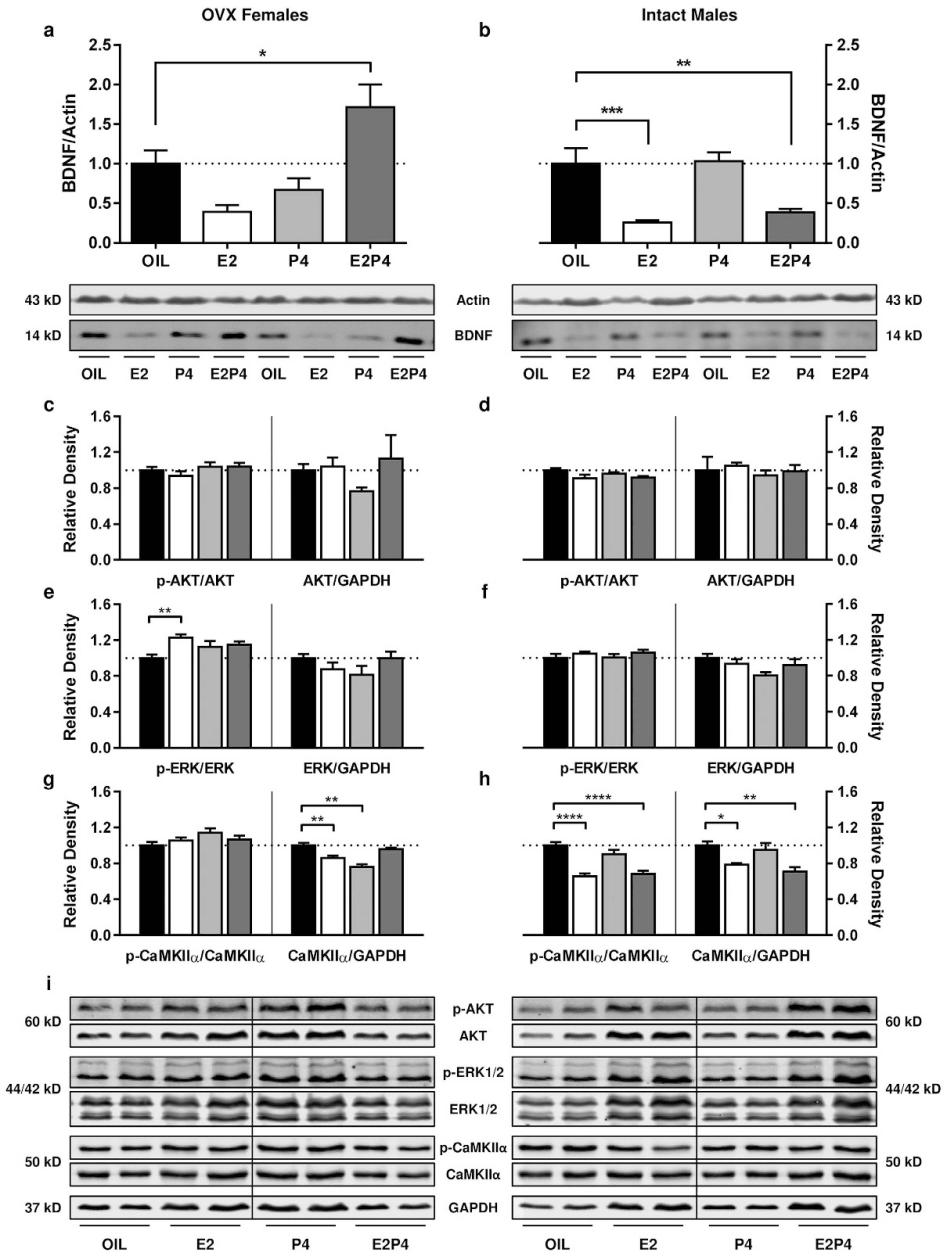
Protein levels of BDNF and downstream signaling effectors 24 h after ketamine in estradiol- and progesterone-treated female and male rats. (**a**) Hippocampal BDNF levels were significantly increased 24 h after low-dose ketamine (2.5 mg/kg, i.p.) only in ovariectomized female rats receiving cyclic treatment with both estradiol and progesterone (E2P4) relative to OIL-treated controls (*p = 0.0345; all other *p* > 0.05). (**b**) BDNF in the hippocampus of intact male rats was decreased 24 h after ketamine in estradiol (E2; ***p = 0.0006) and E2P4-treated (**p = 0.0034) male rats relative to OIL-treated controls, but was unaffected in those receiving progesterone (P4) alone (*p* > 0.05). (**c**,**d**) Neither total nor phosphorylated levels of hippocampal AKT were altered 24 h post-ketamine ketamine in male and female rats regardless of hormone treatment. (**e**,**f**) Phosphorylated ERK1/2 levels were increased following ketamine in E2-treated females (**p = 0.0047), but were otherwise unaffected (*p* > 0.05) in all other treatment conditions. Total ERK abundance was similar between groups, except in P4-treated males which displayed decreased ERK relative to OIL-treated controls (*p = 0.0338). (**g**,**h**) While CaMKIIα phosphorylation was not associated with treatment-response in either sex, lower levels were apparent in E2- (****p < 0.0001) and E2P4-treated (****p < 0.0001) male rats 24 h following ketamine. Parallel decreases in total CaMKIIα levels were observed in the same males relative to OIL-treated counterparts (E2: *p = 0.0196; E2P4: **p = 0.0017). Lower CaMKIIα abundance was also observed at this timepoint in E2- (**p = 0.0033) and P4-treated (****p < 0.0001)—but not E2P4-treated—female rats when compared to same-sex controls. (**i**) Representative western blots for proteins depicted in (**c**,**h**) across all treatment groups. Vertical lines indicate juxtaposition of non-adjacent regions within the same membrane for each phosphorylated and total protein assayed. All data expressed as mean ± SEM (n = 24 female/24 male).

One week following arrival, pair-housed adult female rats began testing in the continuous-access sucrose preference test to determine baseline hedonic behavior. Rats were then ovariectomized (OVX) and allowed one week for recovery before resuming sucrose preference testing. Upon re-stabilization of sucrose preference levels, rats were matched for baseline sucrose preference and body weight and assigned to one of four groups. One group (OVX + E2P4; n = 12) was injected subcutaneously with 2 μg 17-β-estradiol benzoate (E2) in 100 μL sesame oil at 1100 h every fourth day and 500 μg progesterone (P4) in 100 μL sesame oil 24 h later; a second group (OVX + E2; n = 14) received E2 and sesame oil vehicle 24 h later; a third group (OVX + P4; n = 12) received sesame oil vehicle and P4 24 h later; the final group (OVX + OIL; n = 10) received sesame oil vehicle on both days. Every 2 days of injection were followed by 2 days without injection. Hormone doses were chosen based on previous work from our lab^9^, and produce near-physiological levels of E2^41^ and P4^42^ observed throughout a typical 4-day estrous cycle in female rats^43^. The experimental design and hormone treatment regimen are presented in Fig. 1.

Hormone treatments began after habituation to subcutaneous oil injections, and were continued throughout the duration of the experiment. On Day 3 of the third hormone treatment cycle Fig. 1), rats were injected (i.p.) with saline vehicle (SAL), followed 4 d later by 2.5 mg/kg ketamine (KET). SAL and KET were administered 4 h after P4 or oil injections on Day 3 of the treatment cycle to ensure the presence of elevated hormone levels at the time of drug administration. Sucrose preference measurements continued for two additional hormone treatment cycles to examine the duration of KET effects on hedonic behavior.

#### Experiment 1b: Effect of low-dose ketamine on hedonic behavior following cyclic E2 and P4 treatment in intact male rats

Adult pair-housed male rats were tested in the continuous-access sucrose preference test after one week of habituation to the facility to obtain baseline measures of hedonic behavior. Once stable baselines were achieved, rats were matched based on sucrose preference and body weight and assigned to one of four cyclic hormone treatment groups as described above: Intact + E2P4 (n = 10), Intact + E2 (n = 10), Intact + P4 (n = 10), or Intact + OIL (n = 8). Identical experimental procedures and doses of hormone/drug used in Experiment 1 were followed to determine whether cyclic administration of E2 and/or P4 alter behavioral sensitivity of gonad-intact male rats to low-dose ketamine in the sucrose preference test.

#### Experiment 2a: Effect of chronic testosterone treatment on hedonic response to low-dose ketamine in intact female rats

One week following arrival, pair-housed adult female rats began testing in the continuous-access sucrose preference test to determine baseline hedonic behavior. Once stable baselines were achieved, rats were matched by sucrose preference and assigned to one of two groups. One group (Intact + T; n = 10) received a subcutaneous testosterone pellet implant (25 mg/pellet), and a second group (Intact + P; n = 10) received a placebo pellet as a control. Rats were allowed one week for recovery prior to resuming testing. Experimental procedures and drug doses identical to those outlined in Experiment 1 were followed to examine whether activational effects of chronic testosterone treatment influence the hedonic response of intact female rats to low-dose ketamine, except that all groups received oil injections on both days of the hormone treatment cycle instead of E2 and/or P4 to control for injection stress and account for potential effects of oil treatment alone.

#### Experiment 2b: Effect of low-dose ketamine on hedonic behavior following gonadectomy and testosterone supplementation in male rats

One week after habituation to the facility, adult pair-housed male rats began testing in the continuous-access sucrose preference test to determine baseline measures of hedonic behavior. Once stable baselines were achieved, male rats were matched by sucrose preference and body weight and assigned to one of three groups. Two groups were gonadectomized (GDX)—one group (GDX + T; n = 10) received a testosterone pellet implant (25 mg/pellet), and a second group (GDX + P; n = 10) received a placebo pellet implant. A third group (SHAM; n = 10) was sham-operated and implanted with a placebo pellet as a control. Recovery from surgery and all experimental procedures were identical to those described for Experiment 2a. This experiment determined whether gonadal testosterone modulates the effect of low-dose (2.5 mg/kg) ketamine on hedonic behavior in male rats.

### Western blotting

Total proteins were extracted from the same dorsal hippocampus tissue punches used for total RNA isolation (see above) and processed as previously described^9^,^34^,^40^. Immunoblots were blocked in 5% non-fat dry milk in TBS for 1 h at room temperature and incubated at 4 °C overnight with BDNF (Santa Cruz Biotechnology; 1:500), actin (Millipore; 1:5,000), phospho-p44/42^*T202/Y204*^ (Cell Signaling; 1:1,000), p44/42 (Cell Signaling; 1:1,000), phospho-AKT^*S453*^ (Cell Signaling; 1:1,000), AKT (Cell Signaling; 1:1,000), phospho-CaMKII^*T286*^ (Cell Signaling; 1:1,000), or CaMKIIα (6G9) (Cell Signaling; 1:1,000) antibodies. Membranes were washed four times for 5 m each with TBST, then incubated 1 h at room temperature with donkey anti-rabbit IR Dye 680LT (Li-COR Biosciences; 1:10 000) and goat anti-mouse IR Dye 800CW (Li-COR; 1:20 000) fluorescent secondary antibodies. Following four 5-minute TBS washes, membranes were visualized using an Odyssey infrared imaging system (Li-COR Biosciences). Quantification was performed using NIH ImageJ (http://rsbweb.nih.gov/ij). Background-subtracted densities of proteins of interest were normalized to those of either the corresponding total protein for phosphorylated targets, or loading control (actin or GAPDH) for total protein. Normalized data are expressed as fold change relative to control, with control animals set at 1.0.

### Statistical analysis

All data were first subjected to the Anderson-Darling Normality test, and followed a normal distribution. Raw data for sucrose preference, fluid consumption and caloric intake were analyzed by two-way repeated measures analysis of variance (ANOVA). Dunnett’s multiple comparisons tests were then performed where appropriate to determine simple effects of ketamine treatment across time within each hormone condition. Multiplicity-adjusted p-values are reported. Simple linear regression was conducted using SAL baseline sucrose preference as the predictor variable and post-treatment preference scores (expressed as percent change from baseline collapsed across 7 post-treatment days) as the response variable, in order to determine the degree to which baseline hedonic behavior could account for variability in the magnitude of response to KET within each treatment group. Within-group Pearson correlations were used to identify possible associations between daily fluid and caloric intake levels and raw sucrose preference scores following KET treatment. Z-normalized sucrose preference scores and body weight data were analyzed by two-tailed Welsh’s unpaired t-tests or one-way ANOVA, followed by Dunnett’s multiple comparisons tests where appropriate. A detailed description of z-score calculations are presented in Supplementary Materials and Methods online. Comprehensive listings of results from all statistical analyses of behavioral data can be found in Supplementary Tables S1–4 online. Western blot data were analyzed by one-way ANOVA, followed by Dunnett’s tests where appropriate. Alpha was set to 0.05 for all statistical analyses.

## Additional Information

**How to cite this article**: Saland, S. K. *et al*. Hedonic sensitivity to low-dose ketamine is modulated by gonadal hormones in a sex-dependent manner. *Sci. Rep.* **6**, 21322; doi: 10.1038/srep21322 (2016).

## Supplementary Material

Supplementary Table 1

Supplementary Table 2

Supplementary Table 3

Supplementary Table 4

Supplementary Information
