# Supplementary material for: Hedonic sensitivity to low-dose ketamine is modulated by gonadal hormones in a sex-dependent manner
Source: Sci Rep. 2016 Feb 18;6:21322. doi: 10.1038/srep21322 (PMC4766854; doi:10.1038/srep21322)
Supplement: Supplementary Information [file srep21322-s5.pdf]

Supplementary Information

Hedonic sensitivity to low-dose ketamine is modulated by gonadal hormones in a sex dependent manner

Samantha K. Saland, Kristin J. Schoepfer and Mohamed Kabbaj*

Affiliations: Department of Biomedical Sciences & Program in Neuroscience, College of Medicine, Florida State University

*Corresponding author: Mohamed Kabbaj, PhD; Professor, Department of Biomedical Science & Program in Neuroscience, College of Medicine, Florida State University, 1115 W Call Street, Tallahassee, FL, 32306. Email: mohamed.kabbaj@med.fsu.edu; Tel: 850-644-4930.

Supplementary Materials and Methods

Sucrose Preference Z-Score Calculation

Z-score normalization of sucrose preference scores was performed in order to standardize measurements acquired at different times from independent cohorts of rats, thereby allowing direct comparison of the magnitude of treatment (hormone and ketamine) effects on hedonic behavior across experiments—both within and between sexes. Based on methods introduced by Guilloux and colleagues (2012), this approach accounts for non-uniformity of variances between experimental cohorts and across time by normalizing individual measurements to the population mean and standard deviation. Using the following equation, z-scores reflect how many standard deviations (σ) an observation (X) is above or below the mean of a control group (μ) [1]:

$$z = \frac{X - \mu}{\sigma}$$

Here, X represents an individual 24-h sucrose preference score obtained during the post-treatment period. μ and σ represent the mean and standard deviation, respectively, of sucrose preference scores of a designated control group collapsed across all days of the post-treatment period. OIL-treated OVX female and intact male rats were defined as control groups to compare the effects of hormone treatment on hedonic response to ketamine within each sex, whereas OIL-treated OVX female rats served as controls for comparison across both sexes. Individual sucrose preference scores were first expressed as percent change from baseline (SAL) for each day following ketamine treatment, then averaged across the post-treatment period in order to obtain a single measurement for comparison of differences in the *magnitude* of treatment response between sexes under different hormonal conditions. These data were then transformed into z-scores by subtracting the control group mean from individual values, and dividing this difference by the control group standard deviation. Control group values used for standardization represent the mean and standard deviation of preference scores collapsed across the post-treatment period.

Supplementary Figures

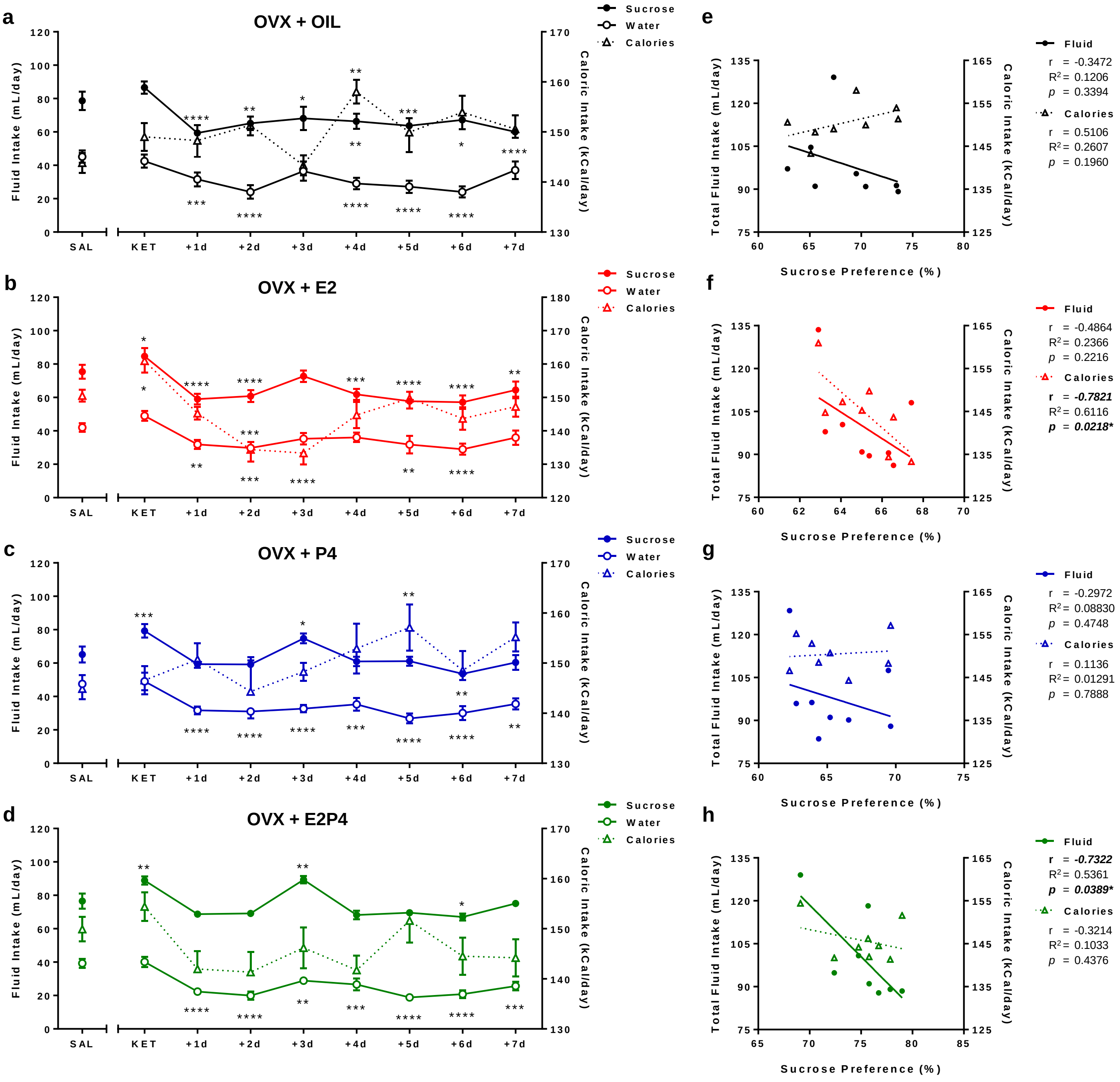

Supplementary Figure S1. Analysis of fluid and caloric intake fluctuations following ketamine treatment in ovariectomized female rats. (a-d) Data are expressed as mean ± SEM (n = 48). Similar changes in sucrose, water and caloric intake following a single low-dose of ketamine (KET; 2.5 mg/kg) in OIL-, estradiol- (E2), progesterone- (P4) and E2P4-treated ovariectomized (OVX) female rats (****$p < 0.0001$, ***$p < 0.001$, **$p < 0.01$, *$p < 0.05$ vs. SAL). **(e-h)** Total fluid and caloric intake were not associated with sucrose preference scores across each day of

the post-treatment period in OIL- and P4-treated OVX female rats. Correlations were observed between sucrose preference scores and caloric intake in OVX+E2 female rats ($r = -0.7821$, $p = 0.0218$) and total fluid intake in OVX+E2P4 female rats ($r = -0.7322$, $p = 0.0389$); however, these factors were negatively associated with sucrose preference, suggesting that the pro-hedonic like effect of KET observed in OVX+E2P4 female rats cannot be explained by drug-induced changes in general consummatory behavior. See Supplementary Table S1 online for a complete report of statistical analyses.

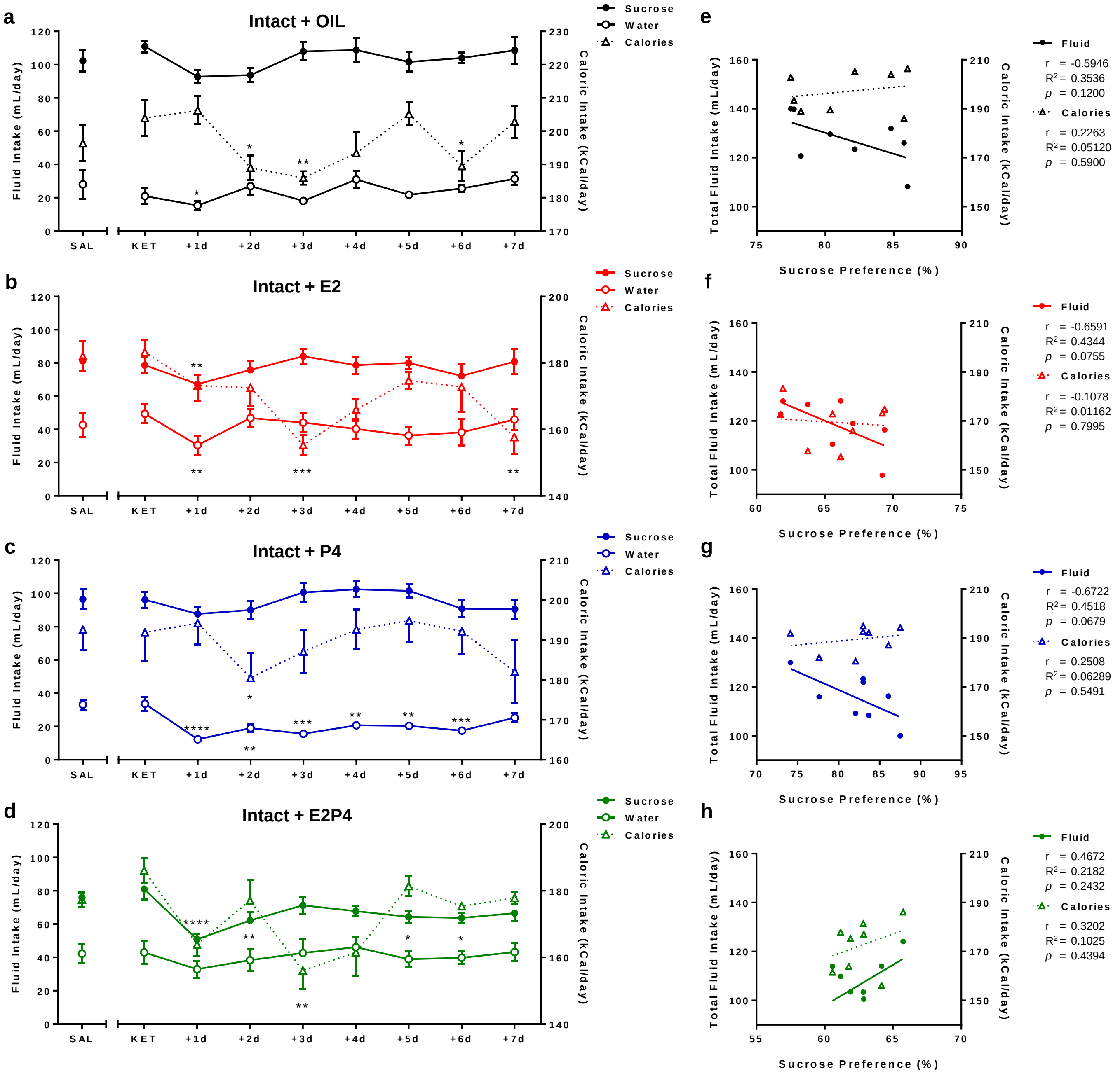

Supplementary Figure S2. Analysis of fluid and caloric intake fluctuations following ketamine treatment in intact male rats. (a-d) Data are expressed as mean ± SEM (n = 40). Changes in sucrose, water and caloric intake following a single low-dose of ketamine (KET; 2.5 mg/kg) in OIL-, estradiol- (E2), progesterone- (P4) and E2P4-treated intact male rats (*****p* < 0.0001, ****p* < 0.001, ***p* < 0.01, **p* < 0.05 vs. SAL). **(e-h)** Total fluid and caloric intake were not associated with sucrose preference scores across each day of the post-treatment period in any group of intact males, suggesting that the enhanced hedonic sensitivity of P4-treated intact male rats to low-dose KET cannot be explained by drug-induced changes in general consummatory behavior. See Supplementary Table S2 online for a complete report of statistical analyses.

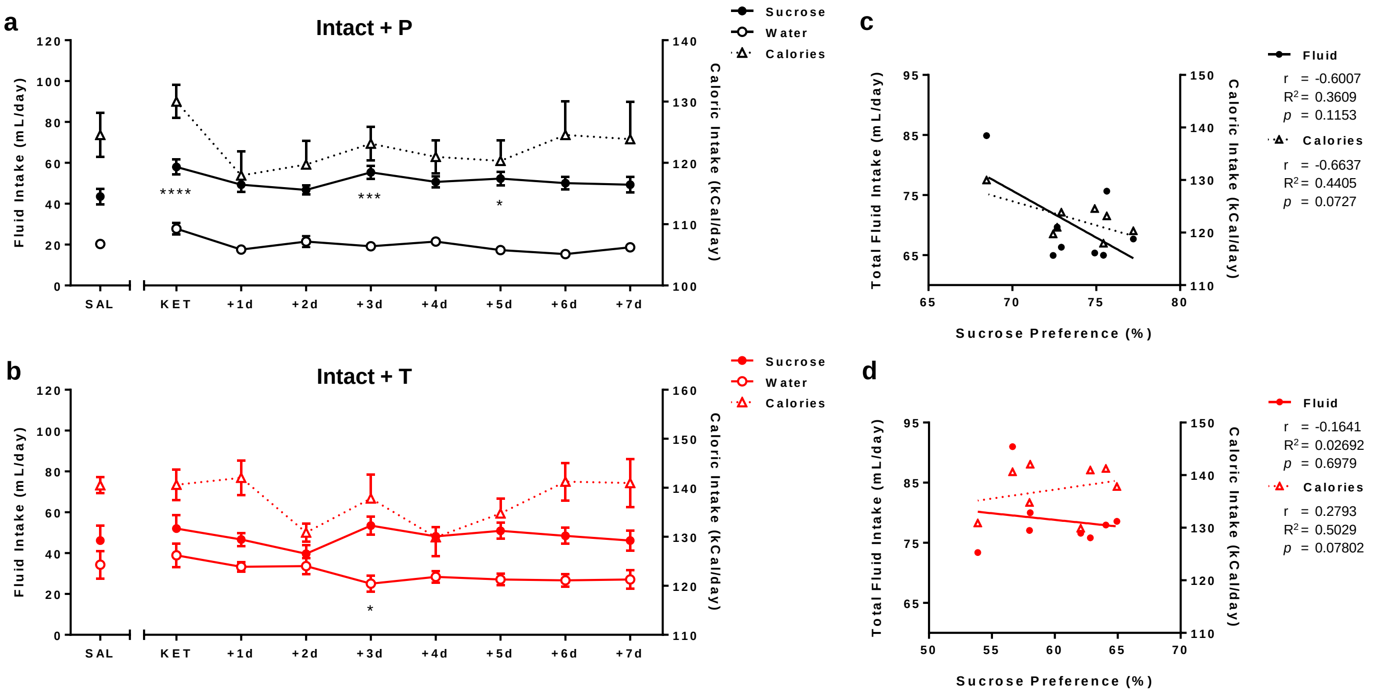

Supplementary Figure S3. Analysis of fluid and caloric intake fluctuations following ketamine treatment in intact female rats. (a-b) Data are expressed as mean ± SEM (n = 18). Changes in sucrose, water and caloric intake following a single low-dose of ketamine (KET; 2.5 mg/kg) in testosterone- (T) and placebo- (P) treated intact female rats (****$p < 0.0001$, ***$p < 0.001$, *$p < 0.05$ vs. SAL). **(c-d)** Total fluid and caloric intake were not associated with sucrose preference scores across each day of the post-treatment period in either T- or P-treated intact female rats, suggesting that the enhanced hedonic sensitivity of normally cycling female rats to low-dose KET cannot be explained by drug-induced changes in general consummatory behavior. See Supplementary Table S3 online for a complete report of statistical analyses.

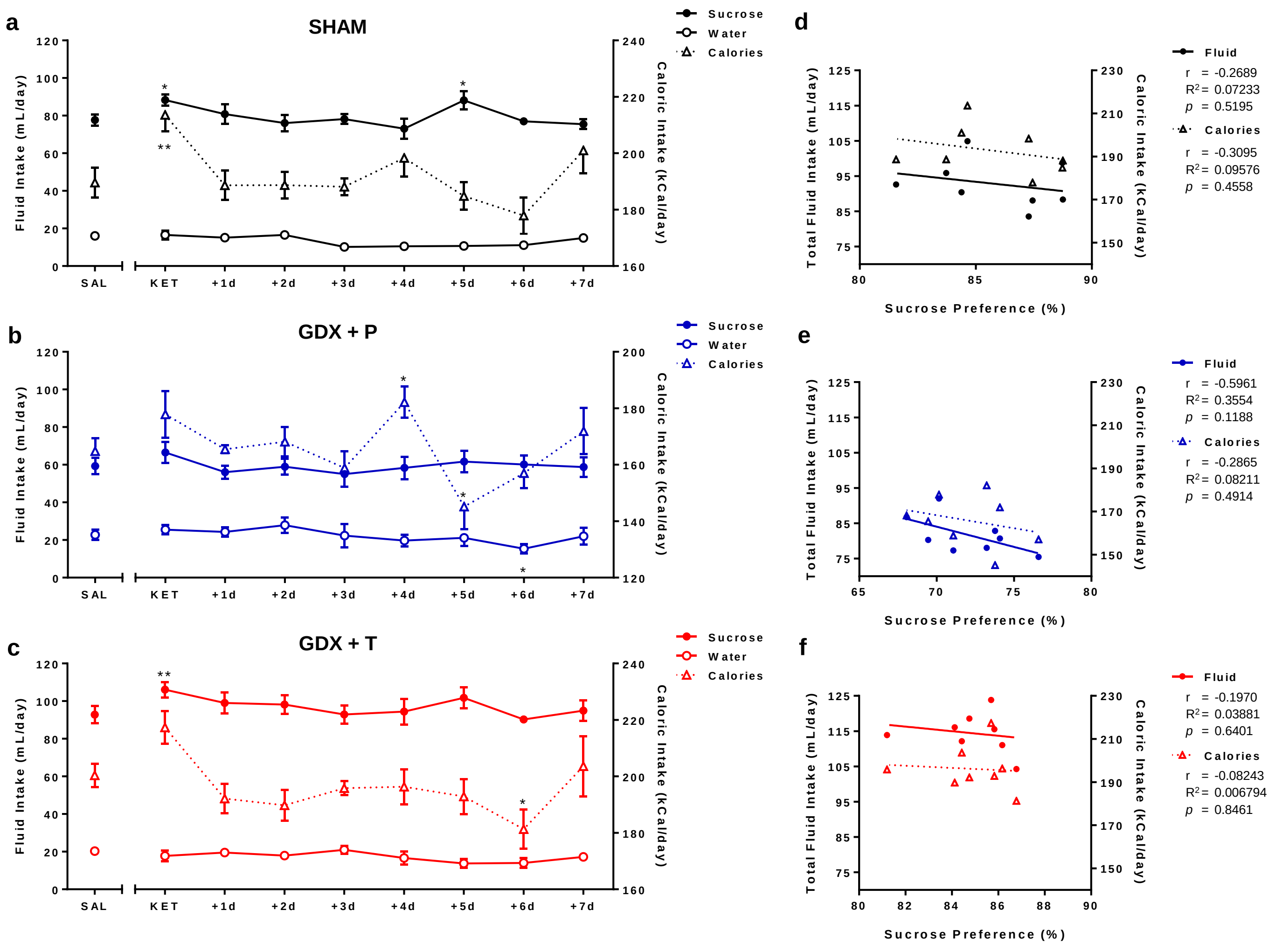

Supplementary Figure S4. Analysis of fluid and caloric intake fluctuations following ketamine treatment in sham-operated and gonadectomized male rats. (a-c) Data are expressed as mean ± SEM (n = 30). Changes in sucrose, water and caloric intake following a single low-dose of ketamine (KET; 2.5 mg/kg) in sham-operated (SHAM) and gonadectomized (GDX) male rats receiving placebo (GDX+P) or testosterone (GDX+T) pellet implants (**$p < 0.01$, *$p < 0.05$ vs. SAL). **(d-f)** Total fluid and caloric intake were not associated with sucrose preference scores across each day of the post-treatment period regardless of circulating testosterone levels, suggesting KET-induced changes in general consummatory behavior cannot explain the lack of response of SHAM or GDX male rats to low-dose KET. See Supplementary Table S4 online for a complete report of statistical analyses.

Supplementary References

1. Guilloux, J.-P., Seney, M., Edgar, N. & Sibille, E. Integrated behavioral z-scoring increases the sensitivity and reliability of behavioral phenotyping in mice: Relevance to emotionality and sex. *J. Neurosci. Methods* **197,** 21–31 (2011).
